# Self-compassion in chronic pain: Validating the self-compassion scale short-form and exploring initial relationships with pain outcomes

**DOI:** 10.1177/20494637241312070

**Published:** 2025-01-07

**Authors:** Jenna L Gillett, Arman Rakhimov, Paige Karadag, Kristy Themelis, Chen Ji, Nicole KY Tang

**Affiliations:** 1 Department of Psychology, 117138 University of Warwick, Coventry, UK; 2 International School of Economics, M. Narikbayev KAZGUU University, Astana, Kazakhstan; 3 School of Arts and Social Sciences, Narxoz University, Almaty, Kazakhstan; 4 Clinical Trials Unit, 117138 University of Warwick, Coventry, UK

**Keywords:** Chronic pain, self-compassion, psychometrics, exploratory structural equation modelling, factor analyses

## Abstract

**Objectives:**

Validate the English version of the *Self-Compassion Scale Short-Form* (SCS-SF) as a reliable measure in chronic pain. Explore self-compassion’s relationship with pain-related outcomes.

**Methods:**

A total of 240 chronic pain patients (at 6-months) and 256 community participants (at 12-months) completed two prospective survey studies. SCS-SF psychometric properties were evaluated through exploratory and confirmatory factor analyses (EFA and CFA), exploratory structural equation modelling (ESEM), test–retest reliability (Pearson’s r) and internal consistency (Cronbach’s α) in both samples. Convergent validity/clinical relevance was assessed in the chronic pain sample via univariate linear regressions between self-compassion and pain intensity, interference, catastrophizing, self-efficacy, anxiety and depression.

**Results:**

The SCS-SF showed acceptable internal consistency in both samples (α > 0.70, range = 0.74–0.79), high test–retest reliability over 6-months in the pain sample (r = 0.81, *p* < .001) and sub-threshold over 12-months in the community (r = 0.59 *p* < .001). EFA revealed a two-factor model distinguishing compassionate and uncompassionate self-responding in both samples. CFA identified a one-factor and two-factor model in both samples, but it did not meet statistical thresholds. ESEM identified the best fit for the chronic pain group was for a two-factor model (RMSEA and SRMR < 0.08; CFI and TLI > 0.90), whereas no models met acceptable fit criteria in the community group. A two-bifactor Bayesian model had suitable fit in both groups. In the chronic pain sample, SCS-SF and compassionate self-responding negatively predicted pain intensity, interference, anxiety, depression, catastrophizing and positively predicted self-efficacy over 6-months. Uncompassionate self-responding positively predicted anxiety, depression, catastrophizing and negatively predicted self-efficacy but did not predict pain outcomes.

**Discussion:**

The SCS-SF is a reliable and valid measure in chronic pain. Total and sub-factor scores appear to have distinct relationships with pain outcomes. Future research should consider self-compassion as a unitary and/or bifactorial (consisting of compassionate and uncompassionate self-responding) construct in chronic pain when measured using the SCS-SF.

## Introduction

Self-compassion can be defined as being inwardly kind to oneself in the face of pain or failure^1,2^ and has been associated with improved psychological outcomes in chronic pain.^3–6^ According to Neff, self-compassion is the extent one embodies self-kindness (vs self-criticism), common humanity (vs isolation) and mindfulness (vs over-identification).^7–9^ These components mutually interact to create a self-compassionate frame of mind that can be operationalised using psychometric scales. Cross-sectional research postulates that higher levels of self-compassion are associated with better adjustment to chronic illness^10,11^ and can improve quality of life in patient populations.^12,13^ Individuals with chronic pain who undergo a self-compassion intervention to cultivate these components report experiencing reduced pain-related fear and disability, as well as improvements in depression, pain acceptance and increased utilisation of coping strategies post-intervention.^14^ Intervention programmes such as compassion-focused therapy^15,16^ and self-compassion training^4,17,18^ are emerging as novel approaches for improving mental health, pain and quality of life in people living with chronic illness where pain is a persistent symptom.^11,19–21^ In chronic pain specifically, cognitive behavioural therapy (CBT) is a recommended psychological approach for improving patient outcomes.^22^ CBT can include elements such as cognitive reframing, pacing and behavioural activation, with specific application to pain,^23,24^ whereas compassion-based interventions build upon cognitive processes^25^ while also cultivating compassion through compassionate imagery exercises and developing an understanding of human regulatory systems.^15,26^ In response to evidence for compassion-based training and therapeutic models, specialised ‘self-compassion for chronic pain’ interventions^5,6,27–29^ have been recently developed as an additional approach to pain care.^5,6^ Furthermore, evidence from an RCT study in *N* = 123 adults with chronic pain highlights the positive impact of mindful self-compassion (*N* = 62) and CBT (*N* = 61) interventions in improving psychological outcomes.^30^ In this study, participants with chronic pain were randomly assigned to a group intervention that entailed 8 × 150-min sessions of either (i) a mindful self-compassion programme or (ii) CBT programme. Each intervention contained standardised content based on (i) the Mindful Self-Compassion protocol^31^ and (ii) Kovacs and Moix’s manual for CBT.^32^ Participant characteristics/baseline measures were not significantly different in each arm. The RCT demonstrated that the self-compassion intervention was more effective than the CBT intervention to increase self-compassion (Average Treatment Effect [ATE] = 0.13, *p* < .05), lower pain interference (ATE = −0.39, *p* < .05) and lower anxiety (ATE = −0.90, *p* < .05).^30^ However, these findings require further investigation and scientific replication in order to fully understand the mechanisms that underpin the relationship between self-compassion and chronic pain.

Much of the literature investigates self-compassion in pain by evaluating the efficacy of self-compassion-based interventions^5,17,33,34^ or utilising cross-sectional assessments^3,35^ to explore relationships between self-compassion and clinical outcomes – while cohort data, across longer time-frames, is generally lacking.^36^ Of the studies mentioned above, psychological measurement of self-compassion predominantly utilises the *Self-Compassion Scale* (SCS)^37^ and its shortened counterpart the *Self-Compassion Scale* *Short-Form* (SCS-SF),^2^ which comprise 26- and 12-items, respectively.^38^ Both measure the six subscales of self-compassion posited by Neff (self-kindness, self-criticism, common humanity, isolation, mindfulness and over-identification). The SCS shows good internal consistency (Cronbach’s α = 0.92), high test–retest reliability after 3 weeks (*r* = 0.93) and convergent validity (e.g. negative associations with self-criticism and a positive association with a sense of social connectedness) in pain-free populations, cross-culturally.^37,39,40^ Often preferred due to its less-burdensome nature,^41^ the SCS-SF also has good internal consistency (Cronbach’s α = 0.86) and is highly correlated with the long form (*r* = 0.97).^2^ However, reliability and validity are yet to be replicated empirically in a chronic pain sample, despite the growing application and interest in self-compassion’s relationship to chronic pain. Such validation is imperative to ensure research applying self-compassion to chronic pain is reliable, valid and that operationalisation of the construct is accurate in clinical populations. As such, this paper aims to address the current problem of a lack of evidence regarding how self-compassion is measured in people with chronic pain and further explore clinical relevance for pain-related processes, outcomes and mental health prospectively.

Both the SCS and SCS-SF scales have been the subject of scientific discussion in recent years^8,42–49^ as the reproducibility of a global self-compassion construct (comprising the six sub-factors posited by Neff) has varied in research.^50^ Recent findings have found evidence for a two-factor structure, consisting broadly of compassionate self-responding (comprising the positive self-kindness, common humanity and mindfulness items) and uncompassionate self-responding (comprising the self-criticism, isolation and over-identification items).^47,51,52^ While other research has found support for one general self-compassion model, as Neff originally postulates, in the form of a single-bifactor structure.^53–56^ Currently, there is no previous psychometric assessment of self-compassion measures in a pain population specifically. Following previous replication studies’ methodology,^50,57,58^ the present study utilises a dual approach to provide evidence for utilising and interpreting the SCS-SF that may be of relevance to clinicians, pain-specialists and psychological researchers. Its aim is to evidence the psychometric properties, validity and reliability of using the SCS-SF in people living with chronic pain, as well as a non-clinical community for context, via in-depth validation in two prospective cohort studies. Well-accepted psychometric testing methods were used, including exploratory and confirmatory factor analyses (EFA and CFA) to explore the underlying factor structure of the SCS-SF and indicate how many constructs the scale is measuring,^59^ exploratory structural equation modelling (ESEM), which is a robust psychometric method that explores scale dynamics and performance,^60^ internal consistency (Cronbach’s α and inter-item correlations) and test–retest reliability (Pearson’s r) across 6–12 months. As a secondary aim, convergent validity and clinical relevance of self-compassion to chronic pain were tested by examining the extent to which self-compassion predicts subsequent pain processes, outcomes and mental health 6-months later.

## Materials and methods

### Design

This study utilised data from two wider prospective cohort studies. The first sample comprised a group of people living with chronic pain (henceforth ‘PLWCP’) who were assessed at baseline and follow-up (6-months). The second sample was a non-clinical community (henceforth ‘community sample’) who were assessed at baseline and follow-up (12-months). Both samples were recruited for non-interventional wider studies, where self-compassion was a variable of interest.

### Participants

#### PLWCP sample

Participants with chronic pain, based in the UK, were recruited online, via opportunity sampling methods between March 2020 and August 2021 as part of the wider Warwick Study of Mental Defeat in Chronic Pain (‘WITHIN’ Study). Recruitment streams included digital advertisements on social media and participant recruitment sites, peer-led support groups, local National Health Service (NHS) pain clinics/private physiotherapists and at local public engagement events. Participants who were interested in taking part, and provided informed consent, were screened for eligibility through an online screening questionnaire. After applying inclusion and exclusion criteria (Table 1), 299 PLWCP were enrolled in the study. After applying further data checks (removal of cases with multivariate outliers, *N* = 2, and cases lost due to follow-up, *N* = 57), the final sample analysed composed of *N* = 240 (80%) participants with chronic pain. As a sensitivity measure, attrition analyses were conducted via Bayesian t-tests and contingency tables to determine differences in participant characteristics of individuals lost to follow-up (*N* = 57) versus those retained (*N* = 240). All tests revealed evidence to indicate no substantial differences (BF^10^ values all < 1.0) in basic demographics (age, gender, ethnicity, education level and employment status) and pain characteristics (pain duration, pain location, presence of widespread pain and MQS score) in the PLWCP sample. Mean self-compassion scores at baseline were also not substantially different between those lost to follow-up versus those retained (BF^10^ value = 0.17).

**Table 1.** Inclusion and exclusion criteria.

*People living with chronic pain inclusion criteria:*
• Aged between 18 and 65
• Experience chronic non-cancer pain for at least 3 months
• Stable treatment regime for duration of the study
• English-speaking (for understanding and implementing the data collection procedure)
• Living in the UK
• Be able to provide informed consent
*People living with chronic pain exclusion criteria:*
• Have any significant comorbid psychiatric (e.g. psychosis), medical (e.g. coronary heart diseases), neurological (e.g. Alzheimer’s, Parkinson’s and epilepsy) or life-threatening conditions that would impact pain experience, impede the ability to provide informed consent or complete the study
• Have elective surgery or procedures requiring general anaesthetic during the study
• Have participated in another research study using an investigational product in the past 3 months
*Community sample inclusion criteria:*
• Aged 18 or older
• English-speaking (for understanding and implementing the data collection procedure)
• Living in the UK
• Be able to provide informed consent
*Community sample exclusion criteria:*
• Being pregnant (due to potential impact on sleep relevant to the wider study)

Note. The examples given in the inclusion/exclusion criteria are not exhaustive, and participants’ eligibility was assessed on a case-by-case basis by the research team.

#### Community sample

Participants based in the UK were recruited online, via opportunity sampling in response to a study advertisement between May 2019 and July 2020 as part of a wider study investigating self-compassion in community adults.^57^ All participants in the community sample reported no chronic pain. Participants who were interested in taking part, and provided informed consent, were screened for eligibility through an online screening questionnaire. After applying inclusion and exclusion criteria (Table 1), a total of 334 participants were enrolled in the study. After performing further data checks (removal of cases due to missing data > 10%,^61^ *N* = 6; multivariate outliers, *N* = 6; and cases lost due to follow-up, *N* = 66), the final community sample was *N* = 256 (77%) participants. Attrition analyses (Bayesian t-tests and contingency tables) between those lost to follow-up (*N* = 66) versus retained (*N* = 256) showed evidence to indicate no substantial differences (BF^10^ values all < 1.0) in the majority of basic demographics (age, gender, education level and employment status). However, there was strong evidence to suggest a difference (BF^10^ value = 32.06) in ethnicity groupings between those lost to follow-up versus those retained, which is acknowledged further in the limitations section. Mean self-compassion scores at baseline were not substantially different between those lost to follow-up versus those retained (BF^10^ value = 0.15) in the community sample.

### Measures

Below are the measures the two studies had in common that were used by the researchers to evaluate the SCS-SF’s performance. All measures were administered in English.

#### Demographics

Participants’ self-reported demographic information (age, gender, ethnicity, education level and employment status) was recorded in both samples. For the PLWCP, pain aetiology questions were also included to determine pain-related characteristics, including pain duration (measured in years up to a maximum of 30+ years), pain location (via a hot-spot body-map), presence of widespread pain and pain medication score. The presence of widespread pain was calculated based on self-reported pain locations from the body map out of a total of 42 areas^62^ and according to the IASP *Classification of Widespread Pain for the International Classification of Diseases* as ‘pain present in the axial skeleton, above and below the waist and in the left and right sides of the body’.^63^ Pain medication was quantified into a single score by using an adapted version of the Medication Quantification Scale-III.^64^ This validated scoring system was adapted by the research team as part of a wider project and included guidance from a pain consultant and a consultant psychiatrist, to include and reflect current prescription procedures in the UK.

#### Psychological and pain-related variables

All participants completed self-reported measures of self-compassion, anxiety and depression. Only the PLWCP sample completed additional measures of pain intensity, pain interference, pain catastrophizing and pain-related self-efficacy.

#### Self-compassion

The 12-item SCS-SF^2^ was used to measure self-compassion. In the community sample, the 12-items that form the SCS-SF were extracted from original responses to the administered SCS^37^ (26-items) for analysis. The 12-items are identical in their wording and provided the researchers a common measure of self-compassion between the PLWCP and community samples without the need to re-administer additional measurement in the form of the SCS-SF in the community sample. Responses are given on a five-point Likert scale ranging from 1 ‘almost never’ to 5 ‘almost always’. The scale includes items from all six sub-components of self-compassion; self-kindness (e.g. *‘I try to be understanding and patient towards those aspects of my personality I don’t like’*), self-judgment (e.g. *‘I’m disapproving and judgmental about my own flaws and inadequacies’*), common humanity (e.g. *‘I try to see my failings as part of the human condition’*), isolation (e.g. *‘when I’m feeling down*, *I tend to feel like most other people are probably happier than I am’*), mindfulness (e.g. *‘when something painful happens I try to take a balanced view of the situation’*) and over-identification (e.g. *‘when I fail at something important to me I become consumed by feelings of inadequacy’*). Negative items are reverse-scored before calculating a total mean SCS-SF score (range: 1-5) whereby a higher score indicates higher levels of self-compassion. The scale has a Cronbach’s alpha value of greater than 0.86 in multiple samples but has not been tested in people with chronic pain specifically.^2^

#### Anxiety and depression symptoms

In the PLWCP sample, the Hospital Anxiety and Depression Scale (HADS) was used as a clinical measure of anxiety and depression symptoms.^65^ The measure contains 14-items, seven depicting anxiety symptoms and seven depicting depression symptoms. Responses are given on a four-point Likert scale (0–3) generating an independent score for anxiety and depression (range: 0-21). Higher scores are indicative of greater symptom severity. A review of the reliability of the HADS demonstrated the anxiety and depression scales have a mean Cronbach’s alpha value of 0.83 and 0.82, respectively, indicating good internal consistency from multiple studies,^66^ including pain samples.^67,68^

In the community sample, the Patient-Reported Outcomes Measurement Information System (PROMIS) Emotional Distress-Anxiety Short-Form and the PROMIS Emotional Distress-Depression Short-Form were used to assess anxiety and depression symptoms. These measures ask participants to assess their feelings/symptoms via ratings to a variety of statements. Responses are made on a five-point Likert scale between 1 ‘never’ and 5 ‘always’. Total raw scores range 8-20, with higher scores representing higher levels of anxiety and/or depression, respectively. Total raw scores convert to T-scores (range: 40.3–81.6 for anxiety and 41.0–79.4 for depression).^69^ The PROMIS scales for anxiety and depression have been reported as reliable and valid for use in research^69^ with Cronbach’s alpha values ranging from 0.89-0.95 and 0.93-0.96, respectively.^69–71^

#### Pain-specific measures (PLWCP sample only)

Pain intensity and pain interference were measured using the Brief Pain Inventory-Short Form (BPI-SF), which is a concise measure widely used in chronic pain research.^72^ The interference subscale contains seven items to assess pain interference levels on an individuals’ general activity, mood, walking ability, work, relationships, sleep and enjoyment of life. Responses are given on a numerical rating scale from 0 ‘does not interfere’ to 10 ‘interferes completely’. Average pain interference is then calculated (range: 0–10), with higher scores indicative of greater levels of pain interference. The pain intensity subscale contains four pain-rating items (‘worst’, ‘least’, ‘average’ and ‘now’) to assess the degree of pain intensity on a numerical rating scale from 0 ‘no pain at all’ to 10 ‘pain as bad as you can imagine’. An average pain intensity score is then calculated (range: 0–10), with higher scores indicative of greater pain intensity. The internal consistency for the BPI-SF ranges 0.89-0.92 for pain interference and 0.80-0.87 for pain intensity.^72^

The tendency to catastrophize about pain was measured using the 13-item Pain Catastrophizing Scale (PCS).^73^ Items are scored on a five-point Likert scale, where 0 = ‘not at all’ and 4 = ‘all the time’, generating a total score (range: 0-52), where higher scores indicate higher levels of pain catastrophizing. The Cronbach’s alpha for the PCS ranges 0.87-0.93, indicating good internal consistency.^73^

Pain-related self-efficacy was measured using the 10-item Pain Self-Efficacy Questionnaire (PSEQ).^74^ Items are scored on a seven-point Likert scale, where 0 = ‘not at all confident’ and 6 = ‘completely confident’, generating a total score (range: 0-60) whereby a higher score indicates higher levels of pain self-efficacy. The Cronbach’s alpha for the PSEQ is 0.92, indicating good internal consistency.^55^

### Study procedures

Participants in both samples underwent a self-report screening questionnaire (after providing informed consent via a consent form) administered online to determine eligibility (see Table 1 for criteria). For the PLWCP group, the screening questionnaire also included open-ended questions to self-report any potential psychiatric, medical and/or neurological conditions for the purposes of meeting study inclusion/exclusion criteria (Table 1). Screening procedures were anonymous and contact details were collected and stored separately for the purpose of follow-up, which participants consented to. If a participant indicated they experienced a condition/symptom that would potentially impact on either their pain experience as part of the disease process or impede ability to give consent or participate in the study, they were excluded. If further details were required, a member of the research team was able to clarify information via email or telephone call where appropriate. All participants were informed of eligibility outcome via email by a member of the research team; if participants were not eligible, they were thanked for their interest and signposted to relevant NIHR research participation webpages. Participants who were eligible were invited to complete the main online survey at baseline and follow-up with informed consent taken via a consent form at each assessment point. Time between assessment points was 6-months (+/− 2 wks) for the PLWCP sample and 12-months (+/− 3 wks) for the community sample in line with study protocols. After completion of each survey, participants were given a short debrief with signposting to various support resources. All participants were reimbursed a £5 voucher for each completed survey.

### Ethical considerations

Ethical approval was granted separately for the two samples analysed in this study, both of which include approval for anonymised data to undergo additional analyses for further research. All analysed data were anonymised using participant ID numbers and were stored on secure servers provided by the University of Warwick. Only approved members of the research team had access to the data.

Ethical approval for data collection for the PLWCP sample comes from part of the wider MRC-funded *WITHIN Study* which was approved by the Health Research Authority and West Midlands – Solihull Research Ethics Committee (Reference Number: 17/WM0053, *p*, IRAS no. 223190). For the community sample ethical approval was obtained separately (as the study was non-clinical in nature) from the Humanities and Social Sciences Research Ethics Committee, University of Warwick, UK (approval number: PGR_18-19/18). The University of Warwick was the sponsor. All research was performed in accordance with the Declaration of Helsinki.

### Statistical analyses

Mplus (Version 8.6) was used for ESEM and CFA, whereas SPSS (Version 28) was used for remaining analyses. Analysed samples include only people who completed self-compassion measures at both time-points, given the interest in examining the test–retest reliability of the SCS-SF and its psychometric properties; thus, attrition was handled by case-wise deletion.

#### Descriptives

SPSS was used to calculate descriptive frequencies, percentages, means and standard deviations. Welch’s t-tests (for continuous variables) and chi-square goodness-of-fit tests (for categorical variables) were used to determine differences between groups in demographics. Descriptive statistics to characterise the samples (means and SDs) are presented for anxiety and depression in both groups, whereas pain catastrophizing, pain-related self-efficacy, pain intensity and pain interference are only reported for the PLWCP group.

#### EFA, CFA and ESEM

**Figure 1.**
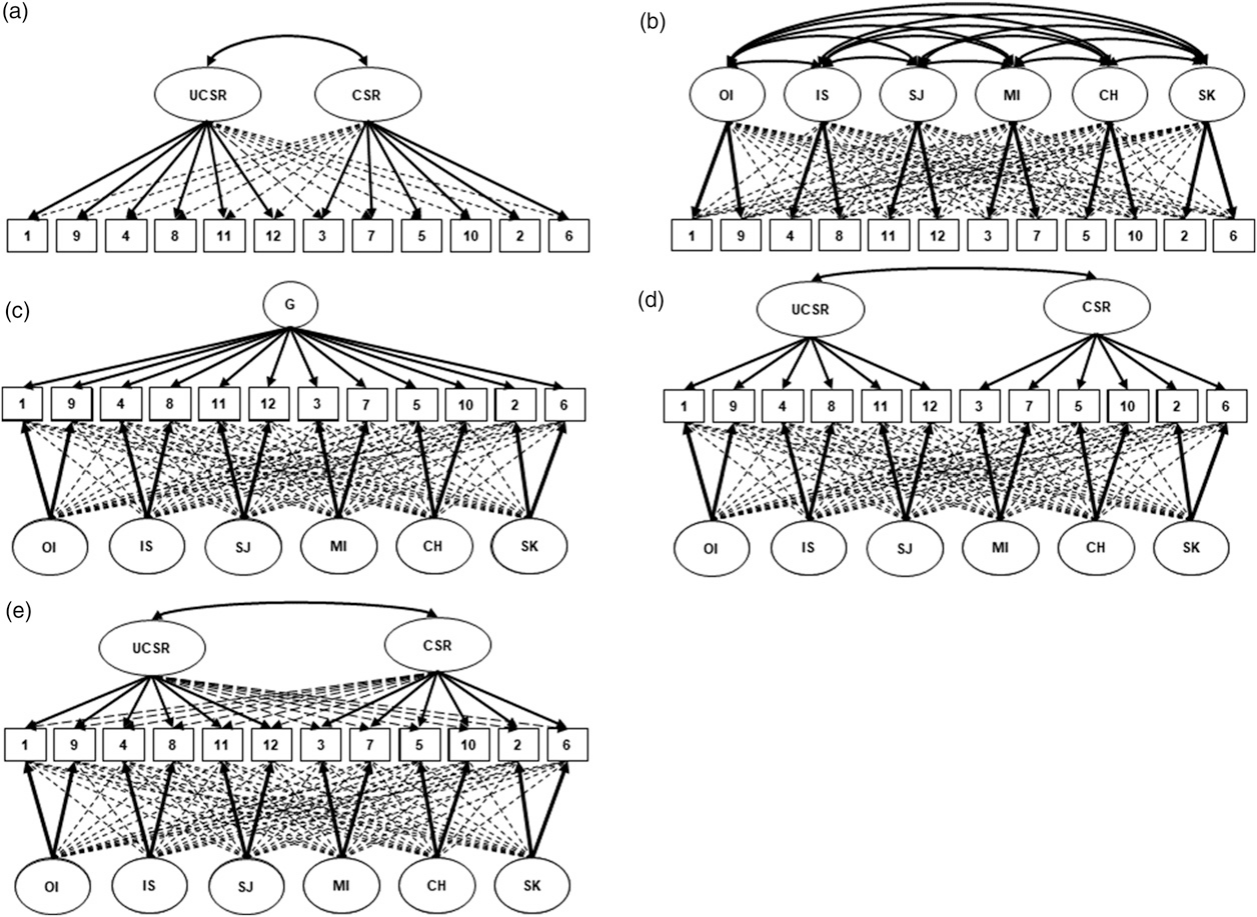
Note. Schematic representation of ESEM model: a two-factor (a), a six-factor (b), a single-bifactor (c) and a two-bifactor (d). Bayes models depict: a Bayes single-bifactor (c) and a Bayes two-bifactor (e). One-factor model is not graphically depicted above. Full arrows indicate target factor loadings; dashed arrows denote cross-loadings. The full arrows are thicker than dashed to ease readers’ perception. G = global factor (total-score). Subscales include the following: SK = self-kindness; SJ = self-judgment; CH = common humanity; IS = isolation; MI = mindfulness; OI = over-identification; CSR = compassionate self-responding; UCSR = uncompassionate self-responding.

Exploratory factor analysis (EFA) can indicate reliability and validity of a measure by observing the factor-structure – that is, exploring how many constructs a scale is measuring and whether a scale measures what it claims to measure. In the original studies, both the SCS^37^ and SCS-SF^2^ posit a single self-compassion factor that comprises the total sum of the six sub-factors (see Figure 1, panel B)^2,9,53,55,58^

Confirmatory factor analyses (CFA) and exploratory structural equation modelling (ESEM) using both the maximum likelihood (ML) and weighted least square mean and variance adjusted (WLSMV) estimation were then conducted. In line with previous replication studies,^8,45,55,57^ this study investigated the following factor-structures: one-factor, two-factor, six-factor, single bi-factor and two-bifactor (see Figure 1). Factor analysis two-sided *p*-values < .05 are considered statistically significant. To replicate previous research, Mplus syntax code from Neff’s study validating the factor-structure of the SCS in 20 diverse (but non-chronic pain) samples^58^ was used. The choice of two estimation methods for determining model fit (ML vs WLSMV) followed previous work^57^ and considered the following statistical argument around appropriate estimations in ESEM. ML usually applies to continuous, normally distributed data, whereas WLSMV is preferred for use with ordinal data. As the SCS-SF responses were answered on a Likert scale, they can be conceptualised as either continuous scores or ordinal; thus, both estimation models were used.^75^ Fit indices applied to all ESEM included: root-mean-square error of approximation (RMSEA) value with 90% confidence intervals (CI), standardised root-mean-square residual (SRMR), Tucker–Lewis index (TLI) and comparative fit index (CFI). An RMSEA and SRMR value of < 0.08, along with a CFI and TLI of > 0.90 (or 0.95), can be considered as an indication of acceptable (or close) model fit.^76,77^ Depending on the estimation method utilised, certain fit indices such as the RMSEA, CFI and TLI have been evidenced to fluctuate substantially^75,78^ hence both ML and WLSMV were used in the present exploratory study. Additionally, competing single-bifactor and two-bifactor models using the Bayes estimator in Mplus were investigated in accordance with recommendations of more recent work^50^ that highlight limitations of two-bifactor models used in older research.^57,58^ For the Bayes estimators, aforementioned fit indices and criteria were applied in addition to the posterior predictive *p*-value (PPP) index, whereby a value < 0.05 indicates a poor fit and a value close to 0.5 indicates a well-fit model.^79^

#### Internal consistency and test–retest reliability

SPSS was used to calculate Cronbach’s alpha values, with an observed cut-off for satisfactory internal consistence (α > 0.7).^80^ Inter-item correlations were also calculated and are available in a matrix for each sample in Supplemental File 1. Pearson’s r correlations for total SCS-SF scores, compassionate and uncompassionate self-responding (CSR; UCSR) sub-scores at baseline and follow-up for both samples assessed test–retest reliability, where r > 0.70 indicates high test–retest reliability.^81^

#### Convergent validity and clinical relevance (PLWCP only)

In the PLWCP sample only, correlations were used in the first instance (see Supplemental File 2) followed by univariate linear regressions to explore prospective relationships between self-compassion and key process/outcome variables (anxiety, depression, pain catastrophizing, pain-related self-efficacy, pain intensity and pain interference) 6-months later as a form of convergent validity and to explore clinical relevance for people with pain.

## Results

### Demographics

Participant’s demographics can be viewed in Table 2. In the PLWCP sample, participants were aged between 18 and 65 years (*M* = 39.9, SD = 12.4), were predominantly female (82.1%) and of Caucasian ethnicity (88.8%). On average participants had experienced pain for 9.6 years (SD = 7.5), and 35.4% had widespread pain. In the community sample, participants were aged between 18 and 85 years (*M* = 37.3, SD = 19.1), were predominantly female (54.7%) and of Caucasian ethnicity (83.2%).

**Table 2.** Participant demographics.

	PLWCP (*n* = 240)	Community sample (*n* = 256)	t or χ^2 ^value
Age^a^ (mean, SD)	39.9 (12.4)	37.3 (19.1)	3.3
Gender (*n*, %)			44.9***
Male	40 (16.7)	114 (44.5)	
Female	197 (82.1)	140 (54.7)	
Other (includes transgender, intersex and non-binary)	3 (1.3)	2 (0.8)	
Ethnicity^b^ (*n*, %)			14.7**
White/Caucasian	213 (88.8)	213 (83.2)	
Black/African/Caribbean/Black British	6 (2.5)	5 (2.0)	
Asian/Asian British	10 (4.2)	31 (12.1)	
Mixed/Multiple ethnicity	11 (4.6)	6 (2.3)	
Other	0	1 (0.4)	
Education (*n*, %)			52.2***
Tertiary	214 (89.2)	160 (62.5)	
Secondary or below	26 (10.8)	79 (30.9)	
Other (not specified)	0	17 (6.6)	
Employment status^c^ (*n*, %)			63.1***
Employed/self-employed	131 (54.6)	108 (42.2)	
Not working	6 (2.5)	4 (1.6)	
Student	70 (29.2)	100 (39.1)	
Retired (incl. medically retired)	19 (17.6)	42 (16.4)	
Other	9 (3.8)	2 (0.8)	
Pain characteristics			
Pain duration in years (mean, SD)	9.6 (7.5)	-	
Pain location^d^ (*n*, %)			
Head	75 (31.3)	-	
Back	153 (63.7)	-	
Shoulder/arm	134 (55.8)	-	
Trunk	124 (51.7)	-	
Hips/buttocks/legs	168 (70)	-	
Widespread pain (*n*, %)	85 (35.4)	-	
Medication score (MQS) (mean, SD)	4.1 (6.6)	-	

Note. Abbreviations and variable information: PLWCP = people living with chronic pain; Tertiary = the educational level following the completion of secondary education; MQS = medication quantification scale. Pain duration was measured in years at screening up to a maximum of 30+ years; pain location was condensed into five categories from the body map index (42 areas total) and participants could have pain in multiple locations; widespread pain, defined as pain present above and below the waist, in the right- and left-sides of the body and in the axial skeleton (as per IASP definitions). Welch’s *t* test (t) for continuous variables and Chi-square goodness-of-fit (χ^2^) for categorical variables were used to test differences between the PLWCP and community samples in demographics. Three variables (gender, ethnicity and employment status) had expected cell counts less than 5. ***p < .001, **p < .01 and *p < .05.

^a^2 people in the PLWCP sample did not disclose their exact age in years (missing).

^b^1 person in the community sample did not disclose their ethnicity (missing).

^c^5 people in each sample did not disclose their employment status (missing).

^d^1 person in the PLWCP sample did not disclose pain locations/have an MQS score (missing).

### Factor analyses and ESEM

To assess the factor structure of the SCS-SF in both samples, factor loadings for each scale item in both samples are presented in Table 3. The outcome of the EFA shows two factors emerged in both samples. One item cross-loaded on both factors (item-6), but the higher loading was for Factor 2 (CSR) so it was included on Factor 2 only. Table 3 also shows the communalities for each item, indicating the proportion of each variable’s variance that can be explained by the two factors. The majority of items met necessary cut-off (≥ 0.05)^61^ apart from item-7 (mindfulness) in both samples, as well as item-6 (self-kindness) and item-8 (isolation) in the community sample. CFA and ESEM analyses confirmed the best fit for the data in the chronic pain sample was for the ESEM two-factor model (Table 4), which had acceptable fit with the ML estimator: RMSEA = 0.08 (90% CI, [0.06–0.10]), CFI = 0.95, TLI = 0.92, SRMR = 0.04. There was no suitable fit for the one-factor, two-factor, six-factor, single bi-factor and two-bifactor models in the community sample.

**Table 3.** EFA item loadings of the SCS-SF in each sample.

			PLWCP (*N* = 240)			Community sample (*N* = 256)		
Item no.	Subscale	Questionnaire item	Factor 1 (CSR)	Factor 2 (UCSR)	*Communalities*	Factor 1 (CSR)	Factor 2 (UCSR)	*Communalities*
1	Over-identification	When I fail at something important to me, I become consumed by feelings of inadequacy		.78	**.66**		.59	**.58**
2	Self-kindness	I try to be understanding and patient towards those aspects of my personality I don’t like	.71		**.57**	.68		**.50**
3	Mindfulness	When something painful happens, I try to take a balanced view of the situation	.79		**.64**	.61		**.42**
4	Isolation	When I’m feeling down, I tend to feel like most other people are probably happier than I am		.64	**.50**		.78	**.60**
5	Common humanity	I try to see my failings as part of the human condition	.78		**.61**	.80		**.66**
6	Self-kindness	When I’m going through a very hard time, I give myself the caring and tenderness I need	.60	.51	**.62**	.61		.38
7	Mindfulness	When something upsets me, I try to keep my emotions in balance	.61		.46	.67		.48
8	Isolation	When I fail at something that’s important to me, I tend to feel alone in my failure		.81	**.69**		.67	.49
9	Over-identification	When I’m feeling down, I tend to obsess and fixate on everything that’s wrong		.75	**.64**		.65	**.64**
10	Common humanity	When I feel inadequate in some way, I try to remind myself that feelings of inadequacy are shared by most people	.68		**.52**	.71		**.51**
11	Self-judgment	I’m disapproving and judgmental about my own flaws and inadequacies		.84	**.74**		.55	**.51**
12	Self-judgment	I’m intolerant and impatient towards those aspects of my personality I don’t like		.73	**.54**		.71	**.55**

Note. Variable information: PLWCP = people living with chronic pain; Community = community sample. Communalities indicate the proportion of variance for that given item that is explained by the factors. Bolded communalities indicate a value ≥ 0.50 (Hair et al., 2010).

**Table 4.** Fit indices (CFA and ESEM) for the SCS-SF in each sample.

		PLWCP (*N* = 240)								Community (*N* = 256)							
		CFA				ESEM				CFA				ESEM			
Estimator	Model	RMSEA [90% CI]	CFI	TLI	SRMR	RMSEA [90% CI]	CFI	TLI	SRMR	RMSEA [90% CI]	CFI	TLI	SRMR	RMSEA [90% CI]	CFI	TLI	SRMR
ML	One-factor	.15 [.13–.16]	.78	.73	.10	Not applicable				.14 [.13–.16]	.73	.67	.10	Not applicable			
	**Two-** **factor**	.09 [.08–.11]	.92	.90	.06	**.08 ** **[.06–.10]**	**.95**	**.92**	**.04**	.11 [.09–.12]	.86	.83	.07	.10 [.08–.12]	.90	.84	.05
	Six-factor	Model was not identified				Model was not identified				Model was not identified				Model was not identified			
	Single-bifactor	Model was not identified				Model was not identified				Model was not identified				Model was not identified			
	Two-bifactor	Model was not identified				Model was not identified				Model was not identified				Model was not identified			
WLSMV	One-factor	.18 [.17–.20]	.85	.82	.09	Not applicable				.18 [.16–.19]	.82	.78	.09	Not applicable			
	Two-factor	.12 [.11–.14]	.94	.92	.05	.10 [.09–.12]	.96	.94	.03	.13 [.12–.15]	.90	.87	.07	.13 [.12–.15]	.92	.88	.05
	Six-factor	Model was not identified				Model was not identified				Model was not identified				Model was not identified			
	Single-bifactor	Model was not identified				Model was not identified				Model was not identified				Model was not identified			
	Two-bifactor	Model was not identified				Model was not identified				Model was not identified				Model was not identified			

Note. Abbreviations and variable information: ML = maximum likelihood estimator; WLSMV = weighted least square mean and variance adjusted estimator; CFA = confirmatory factor analysis; ESEM = exploratory structural equation model; RMSEA = root mean square error of approximation; 90% CI = 90% confidence interval of RMSEA value; CFI = comparative fit index; TLI = Tucker–Lewis index; SRMR = standardised root-mean-square residual; not applicable = not applicable due to no cross-loadings. Bolded rows indicate all acceptable fit criteria are met (RMSEA and SRMR values < 0.08; CFI and TLI values > 0.90).

Examination of single-bifactor and two-bifactor models using the Bayes estimator (Table 5; Figure 1) revealed that the two-bifactor model had a good fit in both PLWCP (RMSEA = 0.02 (90% CI, [0.00–0.05]), CFI = 1.0, TLI = 0.99, PPP = 0.40) and the community sample (RMSEA = 0.02 (90% CI, [0.00–0.04]), CFI = 1.0, TLI = 1.0, PPP = 0.29). The correlation between positive and negative factors was 0.65 (standard error = 0.08 and 0.09, respectively) for both PLWCP and community samples using the Bayes estimator. Standardised factor loadings and reliability estimates for the Bayes two-bifactor model can be found in Supplemental File 3. All items in both samples had meaningful target factor loadings (≥ 0.32)^82^ and corresponding item reliabilities, except isolation item-8 (loading of 0.29) in the community sample. All loadings for specific factors were non-significant, except for isolation item-4 and item-8 (loadings of 0.62 and 0.55, respectively), and self-judgment item-12 (0.17) in the community sample, as well as the self-judgment item-11 and item-12 (0.31 and 0.41) in the pain sample (Supplemental File 3).

**Table 5.** Fit indices Bayes estimator single-bifactor and two-bifactor models.

Model	PLWCP (*N* = 240)							Community (*N* = 256)						
	Free	Chi-OBS	Chi-REP	PPP	RMSEA [90% CI]	CFI	TLI	Free	Chi-OBS	Chi-REP	PPP	RMSEA [90% CI]	CFI	TLI
Single-bifactor	Model was not identified							Model was not identified						
**Two-** **bifactor**	**121**	**−33**	**43**	**.40**	**.02 ** **[.00–.05]**	**1.00**	**.99**	**121**	**−34**	**41**	**.29**	**.02 ** **[.00–.04]**	**1.00**	**1.00**

Note. Abbreviations and variable information: Free = number of free parameters; chi-OBS = chi-square observed; chi-REP = chi-square replicated; PPP = posterior predictive p-value; RMSEA = root mean square error of approximation; 90% CI = 90% confidence interval of RMSEA value; CFI = comparative fit index; TLI = Tucker–Lewis index. Bolded rows indicate all acceptable fit criteria are met (RMSEA and SRMR values <0.08; CFI and TLI values > 0.90, PPP value >.05).

### Internal consistency, test–retest reliability

In line with the EFA, ESEM and CFA results, and in conjunction with further theoretically driven considerations, the two factors were defined in PLWCP as ‘compassionate self-responding’ (CSR; comprising positive subscale items: self-kindness, common humanity and mindfulness) and ‘uncompassionate self-responding’ (UCSR; comprising negative subscale items: self-judgment, isolation and over-identification). Table 6 presents the internal consistency (Cronbach’s α) and test–retest reliability scores for each sample. Internal consistency for all measures of self-compassion (total SCS-SF, CSR and UCSR scores) ranged from 0.74 to 0.90, indicating acceptable internal consistency (α > 0.70) in both samples at baseline and follow-up. Test–retest reliability for all measures of self-compassion were higher in the PLWCP group than in the community sample (PLWCP range: 0.69–0.81; community sample range: 0.51–0.59). In PLWCP, test–retest reliability was acceptable (r > 0.70) over 6-months for total SCS-SF and UCSR scores (r = 0.81 and 0.78, respectively) while CSR score approached acceptability threshold (r = 0.69). For the community sample, none of the scores met the acceptable threshold over 12-months.

**Table 6.** Internal consistency and test–retest reliability statistics.

	PLWCP			Community sample		
	Baseline α	Follow-up α	Test–retest (r)	Baseline α	Follow-up α	Test–retest (r)
SCS-SF score	.89	.90	.81***	.75	.82	.59***
CSR score	.83	.85	.69***	.80	.74	.51***
UCSR score	.88	.88	.78***	.79	.78	.54***

Note. Abbreviations and variable information: SCS-SF = self-compassion scale short-form; CSR = compassionate self-responding; UCSR = uncompassionate self-responding. Total mean scores used. ***p < .001, **p < .01 and *p < .05.

### Convergent validity and clinical relevance

Total mean and standard deviations for SCS-SF, CSR and UCSR scores for both groups, at baseline and follow-up, can be viewed in Table 7 along with key variables of interest for clinical relevance at both time-points for PLWCP. Kendall’s tau-b correlations between each variable were all significant (*p* < .05 or lower) (see Supplemental File 2 for correlation matrix) and warranted univariate linear regressions to determine if total SCS-SF score as well as CSR and UCSR differed in predictivity of pain and mental health processes/outcomes 6-months later. Table 8 shows the standardised regression coefficients (β) for each measure at follow-up predicted by total self-compassion scores at baseline. Total SCS-SF score significantly, positively predicted pain-related self-efficacy and significantly negatively predicting all other variables (*p* < .01). CSR also significantly predicted all outcomes (*p* < .001), in the same directions. UCSR positively predicted anxiety, depression and pain catastrophizing, negatively predicted pain-related self-efficacy (*p* < .05) and did not significantly predict pain intensity and interference.

**Table 7.** Self-compassion and main variable scores.

	PLWCP		Community sample	
	Baseline	Follow-up	Baseline	Follow-up
Self-compassion (predictor variable) (mean, SD)				
SCS-SF score	2.9 (0.8)	2.9 (0.8)	2.9 (0.6)	3.1 (0.6)
CSR score	3.1 (0.8)	3.1 (0.9)	3.2 (0.8)	3.2 (0.6)
UCSR score	3.3 (1.0)	3.2 (1.0)	3.3 (0.9)	2.9 (0.8)
Mental health (mean, SD)				
Anxiety	8.6 (4.9)	8.4 (4.7)	51.0 (10.9)	51.2 (10.7)
Depression	7.8 (4.7)	7.6 (4.9)	49.4 (10.2)	53.0 (8.7)
Pain processes (mean, SD)				
Pain-related self-efficacy	34.3 (14.8)	35.1 (14.9)	-	-
Pain catastrophizing	19.9 (12.7)	21.7 (12.6)	-	-
Pain outcomes (mean, SD)				
Pain intensity rating	4.4 (1.9)	4.3 (2.0)	-	-
Pain interference rating	4.2 (2.1)	4.9 (2.7)	-	-

Note. Abbreviations and variable information: SCS-SF = self-compassion scale short-form; CSR = compassionate self-responding (comprising positive SCS-SF scale items); UCSR = uncompassionate self-responding (comprising negative SCS-SF scale items). Anxiety and depression were measured by the Hospital Anxiety and Depression scale (HADS) in the PLWCP sample and the Patient-Reported Outcomes Measurement Information System (PROMIS) Emotional Distress-Anxiety/Depression Short-Form(s) in the community sample, so were not compared for between-group difference in scores. Pain processes and outcomes were only recorded in the PLWCP sample.

**Table 8.** Prospective univariate linear regression model statistics (6-months) in PLWCP.

	Total SCS-SF		CSR		UCSR	
	F	β	F	β	F	β
Outcome variable						
Anxiety	115.4^***^	−.57	47.52^***^	−.41	121.36^***^	.58
Depression	56.3^***^	−.44	55.27^***^	−.44	32.34^***^	.35
Pain-related self-efficacy	20.7^***^	.28	35.74^***^	.36	6.09^*^	−.16
Pain-catastrophizing	50.5^***^	−.42	37.2^***^	−.37	37.6^***^	.37
Pain intensity	6.8^**^	−.17	15.83^***^	−.25	.98	.06
Pain interference	11.1^***^	−.21	19.06^***^	−.27	3.22	.12

Note. β = Standardised regression coefficient (beta weight). ***p < .001, **p < .01 and *p < .05.

## Discussion

This study explored the psychometric properties, reliability and clinical relevance of one of the most frequently utilised measures of self-compassion, the SCS-SF, in a sample of people with chronic pain and a pain-free community. Factor analyses explored the underlying factor structure of the SCS-SF as well as ESEM methods in both samples, to further evidence scale dynamics and performance. The findings of this study demonstrated evidence for a two-factor and two-bifactor model in PLWCP, and a two-bifactor model in the pain-free community. Following previous literature and a data-driven approach, these factors were conceptualised as ‘compassionate self-responding’ and ‘uncompassionate self-responding’. This structure was partially confirmed via ESEM; only one estimation method supported the two-factor model in the PLWCP sample – which is likely related to the tendency of ML estimation to provide a less biased fit than WLSMV estimation.^75^ The two-bifactor model in turn was fully supported by Bayes estimation in both samples. Internal consistency and test–retest reliability were assessed in both conventional total SCS-SF score as well as separated CSR and UCSR scores in both samples. Higher internal consistency was found in PLWCP, but acceptable α levels occurred in both samples. The community sample had subthreshold test–retest reliability, while the PLWCP group exceeded or approached the cut-off for good test–retest reliability. Following this, clinical relevance of self-compassion in PLWCP and expanded convergent validity were explored by measuring the predictive nature of self-compassion scores. Total SCS-SF and CSR predicted all variables significantly in expected directions at 6-months (i.e. higher self-compassion scores predicted lower scores in anxiety, depression pain catastrophizing, pain intensity and interference and higher scores in pain-related self-efficacy). UCSR significantly predicted anxiety, depression, pain catastrophizing and pain-related self-efficacy in opposite directions but was not a significant predictor of pain outcomes (intensity and interference) at 6-months.

The outcome of the factor analyses and ESEM demonstrate evidence to suggest self-compassion measured by the SCS-SF in people with chronic pain may be best represented by a two-factor model and/or Bayesian two-bifactor model. In the original scale validation studies both the SCS^37^ and SCS-SF^2^ posit a single-factor with six sub-factors – whereby one general self-compassion score combines the six sub-scales (self-kindness, self-criticism, common-humanity, isolation, mindfulness and over-identification).^2,9^ Although the current study’s findings did not support this structure, a two-factor structure to the SCS-SF has been referenced previously in both non-pain clinical samples^44^ and healthy volunteers.^45,83–86^ Also, these findings are consistent with the results of a recent psychometric study into the SCS long-form, wherein a two-bifactor model was supported in an international sample of non-clinical participants.^87^ Thus, this paper contributes to the literature in this area from a representative chronic pain demographic and has provided a new comparison point for similar studies in this area. However, it is important to note the two factors observed could be an artifact of the positively and negatively formulated items, as Neff herself argues both contribute to self-compassion as a whole.^9,54^ Nonetheless, the theoretical underpinnings to the positive and negative components have also been posited as independent from one another; capturing two distinct processes in other research.^45,48,84^ As to whether these two components (CSR and UCSR) are fundamentally mutually exclusive, and therefore may potentially predict different outcomes, further insight is offered below.

In terms of clinical relevance, it was found that self-compassion univariately predicted pain-related processes, outcomes and mental health in people living with chronic pain. Total SCS-SF and CSR scores significantly negatively predicted anxiety, depression, pain-catastrophizing, pain intensity and pain interference as well as positively predicted pain-related self-efficacy 6-months later. This supports previous findings wherein improved anxiety^30^ and depression symptoms^36,88^ have been associated with higher self-compassion scores in people with chronic pain. Additionally, fewer pain-catastrophizing thoughts and increased sense of pain-related self-efficacy have also been associated with higher self-compassion in the literature.^35^ Thus, this study provides preliminary evidence to support the idea that increased self-compassion score (particularly total SCS-SF and CSR) is a protective factor in chronic pain, as has been found previously.^3,5,6,16,36^ The findings also demonstrate that UCSR scores did not predict pain intensity or interference, implying that this negative side to self-compassion may be less salient to those experiencing persistent pain in relation to impacting pain outcomes 6-months later. One reason for this could be that UCSR items may indeed be measuring a latent factor. Other researchers have evidenced the overlap between UCSR and general ‘self-criticism’,^89,90^ and this lends evidence to the argument that UCSR may be tapping into a latent construct. If so, it could potentially explain the lack of predictive relationship between UCSR on 6-month pain outcomes in the present study, as self-criticism has been found to impact the affective, but not sensory (i.e. intensity and interference), components of pain.^91^ Additional evidence exists to support the mutually exclusive predictive nature of UCSR versus CSR in relation to wider psychological variables,^45,89^ and also in disordered eating behaviours.^92^ One study in chronic pain specifically highlights UCSR is a stronger predictor of depression symptoms above and beyond pain intensity and disability,^93^ but this study does not expand on the potential predictive power on pain outcomes themselves. Given this evidence collectively, alongside the present findings, it is possible that the UCSR items of the SCS-SF may best capture a latent variable that is perhaps less relevant to predicting pain outcomes specifically, and this should therefore be considered when applying self-compassion to chronic pain. In the present study, total SCS-SF and CSR were nonetheless found to predict pain intensity and interference, as has been replicated in other work,^33,94,95^ but these effects do not always remain long-term.^33^ Muris’ work^46,49,96^ validating self-compassion measurements (though not in chronic pain) concluded that the negative subscales appear to inflate the negative relationship between self-compassion and psychopathology in particular – which could explain why the pain process and mental health variables were significant in the present study, and not pain intensity and interference outcomes when predicted by UCSR alone. While it remains conventional to use a total SCS-SF score, to better capture the full range of variance than two separate UCSR and CSR scores,^8^ when it comes to pain-related outcomes in particular, considering the unique prediction of UCSR versus CSR separately may be useful to inform and provide context to future research in self-compassion and chronic pain.

This study has several limitations. First, the demographics of both samples consisted of predominantly Caucasian ethnicity, female, highly educated participants and therefore findings from this investigation may limit generalisability. It is worth noting that the pain sample is representative of UK population pain-demographics, which reports that females are most likely to be affected by chronic pain.^97^ Further consideration of how demographics may influence the generalisability of this study relate to attrition in the community sample, which evidenced a difference in ethnicity between those retained versus those lost to follow-up. The current study did not formally control for the demographic differences in the retained community versus PLCWP sample due to the focus of the study primarily being provision of psychometric evidence of the SCS-SF in people with chronic pain. However, the UK has been found to have lower levels of self-compassion than that of other countries.^55^ As the present study was UK-based, it is worth considering the role culture may have in the performance of self-compassion related measures in particular. Cultural differences may, for example, exist in participants’ perceptions and perceived conceptualisations of self-compassion, as has been found previously.^98–100^ Furthermore, cultural differences may exist regarding pain reporting and pain-management strategies^101,102^; thus, it is important to consider this potential confounding influence. The authors recognise the importance of inclusive research and therefore recommend replications in larger and more heterogeneous groups to improve representation of current findings, in more diverse samples, cross-culturally. The long-form SCS administered in the community sample (which contains all the items of the SCS-SF administered in the PLWCP sample) has been found to be widely representative, irrespective of gender, language, or community status. However, the ordering of the 12 scale-items that feature in both scales does differ; for example, item-2 in the SCS-SF appears as item-26 in the SCS. As such, the authors acknowledge the potential impact and difference of item ordering on participants’ user experience and in response to the self-compassion measure between each sample. Furthermore, in collating data for the present study to examine the SCS-SF’s test–retest reliability over time, the follow-up durations were different between the two samples (6-months in PLWCP vs 12-months in the community sample). As such, the findings provide valuable insights into the acceptability of the SCS-SF’s test–retest reliability over 12-months in non-clinical chronic pain-free adults and over 6-months in people with chronic pain. However, it is important to note that any direct comparisons of the SCS-SF’s test–retest reliability in this study between the two samples would be confounded with a duration difference. Future studies investigating the SCS-SF in people with pain would benefit from a longer follow-up duration to add further evidence in this field.

## Conclusion

In response to growing application of self-compassion to chronic pain, this study was one of the first to validate the use of the SCS-SF as a measure of self-compassion in a pain population. The outcome of this research highlights that a two-factor and Bayesian two bi-factor model had acceptable fit for the data in people with pain, while just a Bayesian two bi-factor model had acceptable fit in the non-clinical community sample. Total SCS-SF score and CSR scores predicted key mental health and pain-related processes/outcomes 6 months later, while UCSR did not predict pain outcomes. It is recommended that researchers investigating self-compassion in the context of chronic pain present both a conventional total score for the SCS-SF and supplement this by also considering the individual nature of CSR and UCSR separately, particularly in the context of pain outcomes. More evidence is needed to investigate the relationships and predictivity of how self-compassion may impact other aspects of chronic pain in addition to the six process/outcome variables explored in this study. Nonetheless, the protective nature of cultivating self-compassion seems relevant to psychological literature and should continue to be explored in pain research.

## Supplemental Material

Supplemental Material - Self-compassion in chronic pain: Validating the self-compassion scale short-form and exploring initial relationships with pain outcomes Supplemental Material for Self-compassion in chronic pain: Validating the self-compassion scale short-form and exploring initial relationships with pain outcomes by Jenna L Gillett, Arman Rakhimov, Paige Karadag, Kristy Themelis, Chen Ji and Nicole KY Tang in British Journal of Pain
