# Supplementary material for: Self-compassion in chronic pain: Validating the self-compassion scale short-form and exploring initial relationships with pain outcomes
Source: Br J Pain. 2025 Jan 7;19(4):239–56. doi: 10.1177/20494637241312070 (PMC11707776; doi:10.1177/20494637241312070)
Supplement: Supplemental Material - Self-compassion in chronic pain: Validating the self-compassion scale short-form and exploring initial relationships with pain outcomes [file sj-pdf-1-bjp-10.1177_20494637241312070.pdf]

Supplementary File 1. Inter-item correlation matrices for the SCS-SF in each sample

PLWCP	Item 1 "When I fail at something important to me I become consumed by feelings of inadequacy"	Item 2 "I try to be understanding and patient towards those aspects of my personality I don't like"	Item 3 "When something painful happens I try to take a balanced view of the situation"	Item 4 "When I'm feeling down, I tend to feel like most other people are probably happier than I am"	Item 5 "I try to see my failings as part of the human condition"	Item 6 "When I'm going through a very hard time, I give myself the caring and tenderness I need"	Item 7 "When something upsets me I try to keep my emotions in balance"	Item 8 "When I fail at something that's important to me, I tend to feel alone in my failure"	Item 9 "When I'm feeling down I tend to obsess and fixate on everything that's wrong"	Item 10 "When I feel inadequate in some way, I try to remind myself that feelings of inadequacy are shared by most people"	Item 11 "I'm disapproving and judgmental about my own flaws and inadequacies"	Item 12 "I'm intolerant and impatient towards those aspects of my personality I don't like"
"When I fail at something important to me I become consumed by feelings of inadequacy"												
"I try to be understanding and patient towards those aspects of my personality I don't like"	-0.218***											
"When something painful happens I try to take a balanced view of the situation"	-0.241***	0.433***										
"When I'm feeling down, I tend to feel like most other people are probably happier than I am"	0.394***	-0.228***	-0.332***									
"I try to see my failings as part of the human condition"	-0.182***	0.399***	0.421***	-0.188***								
"When I'm going through a very hard time, I give myself the caring and tenderness I need"	-0.403***	0.444***	0.365***	-0.374***	0.408***							
"When something upsets me I try to keep my emotions in balance"	-0.289***	0.379***	0.426***	-0.272***	0.321***	0.412***						
"When I fail at something that's important to me, I tend to feel alone in my failure"	0.538***	-0.267***	-0.211***	0.494***	-0.175**	-0.439***	-0.243***					
"When I'm feeling down I tend to obsess and fixate on everything that's wrong"	0.531***	-0.232***	-0.231***	0.441***	-0.233***	-0.387***	-0.349***	0.515***				
"When I feel inadequate in some way, I try to remind myself that feelings of inadequacy are shared by most people"	-0.306***	0.42***	0.388***	-0.237***	0.408***	0.374***	0.254***	-0.312***	-0.328***			
"I'm disapproving and judgmental about my own flaws and inadequacies"	0.539***	-0.31***	-0.167**	0.346***	-0.173**	-0.436***	-0.307***	0.541***	0.534***	-0.274***		
"I'm intolerant and impatient towards those aspects of my personality I don't like"	0.379***	-0.278***	-0.088	0.340***	-0.139**	-0.373***	-0.187***	0.411***	0.381***	-0.192***	0.564***	

Note. Based on baseline SCS-SF scores. Kendall's Tau-b correlations applied. Correlation coefficient cut-offs: + or - 0.10: very weak. + or -0.10 to 0.19: weak. + or - 0.20 to 0.29: moderate. + or - 0.30 or above: strong.
***p<.001, **p<.01, *p<.05

Colour Key

-1 -0.5 0 0.5 1

COMMUNITY SAMPLE	Item 1 "When I fail at something important to me I become consumed by feelings of inadequacy"	Item 2 "I try to be understanding and patient towards those aspects of my personality I don't like"	Item 3 "When something painful happens I try to take a balanced view of the situation"	Item 4 "When I'm feeling down, I tend to feel like most other people are probably happier than I am"	Item 5 "I try to see my failings as part of the human condition"	Item 6 "When I'm going through a very hard time, I give myself the caring and tenderness I need"	Item 7 "When something upsets me I try to keep my emotions in balance"	Item 8 "When I fail at something that's important to me, I tend to feel alone in my failure"	Item 9 "When I'm feeling down I tend to obsess and fixate on everything that's wrong"	Item 10 "When I feel inadequate in some way, I try to remind myself that feelings of inadequacy are shared by most people"	Item 11 "I'm disapproving and judgmental about my own flaws and inadequacies"	Item 12 "I'm intolerant and impatient towards those aspects of my personality I don't like"
"When I fail at something important to me I become consumed by feelings of inadequacy"												
"I try to be understanding and patient towards those aspects of my personality I don't like"	-0.306***											
"When something painful happens I try to take a balanced view of the situation"	-0.314***	0.321***										
"When I'm feeling down, I tend to feel like most other people are probably happier than I am"	0.257***	-0.168**	-0.169**									
"I try to see my failings as part of the human condition"	-0.401***	0.436***	0.465***	-0.109*								
"When I'm going through a very hard time, I give myself the caring and tenderness I need"	-0.244***	0.39***	0.226***	-0.072	0.324***							
"When something upsets me I try to keep my emotions in balance"	-0.313***	0.373***	0.47***	-0.175**	0.390**	0.295***						
"When I fail at something that's important to me, I tend to feel alone in my failure"	0.143**	-0.037	-0.052	0.364***	0.055	0.010	0.031					
"When I'm feeling down I tend to obsess and fixate on everything that's wrong"	0.483***	-0.328***	-0.322***	0.352***	-0.346***	-0.222***	-0.408***	0.128**				
"When I feel inadequate in some way, I try to remind myself that feelings of inadequacy are shared by most people"	-0.242***	0.339***	0.314***	-0.005	0.425***	0.311***	0.292***	0.032	-0.247***			
"I'm disapproving and judgmental about my own flaws and inadequacies"	0.531***	-0.338***	-0.255***	0.190***	-0.353***	-0.164**	-0.251***	0.043	0.542***	-.222***		
"I'm intolerant and impatient towards those aspects of my personality I don't like"	0.386***	-0.296***	-0.217***	0.359***	-0.212***	-0.231***	-0.244***	0.214***	0.422***	-0.094	0.388***	

*Note. Based on baseline SCS-SF scores. Kendall's Tau-b correlations applied. Correlation coefficient cut-offs: + or - 0.10: very weak. + or -0.10 to 0.19: weak. + or - 0.20 to 0.29: moderate. + or - 0.30 or above: strong. ***p<.001, **p<.01, *p<.05*

Colour Key

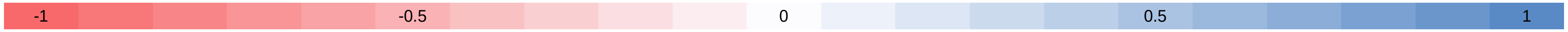

	Self-compassion			Mental Health		Pain Processes		Pain Outcomes	
	Total SCS-SF	*CSR*	*UCSR*	*Anxiety*	*Depression*	*Pain-related self-efficacy*	*Pain catastrophi-zing*	*Pain intensity*	*Pain interference*
Total SCS-SF									
CSR	0.683***								
UCSR	-0.764***	-0.414***							
Anxiety	-0.452***	-0.343***	0.439***						
Depression	-0.383***	-0.365***	0.319***	0.484***					
Pain-related self-efficacy	0.228***	0.284***	-0.13**	-0.301***	-0.527***				
Pain catastrophizing	-0.333***	-0.295***	0.306***	0.423***	0.451***	-0.401***			
Pain intensity	-0.073*	-0.109**	0.031*	0.249***	0.325***	-0.365***	0.254***		
Pain interference	-0.137**	-0.121**	0.116*	0.169***	0.307***	-0.283***	0.199***	0.355***	

*Note. Based on baseline scores. Kendall's Tau-b correlations applied. Correlation coefficient cut-offs: + or - 0.10: very weak. + or -0.10 to 0.19: weak. + or - 0.20 to 0.29: moderate. + or - 0.30 or above: strong. ***p<.001, **p<.01, *p<.05*

-1 -0.5 0 0.5 1 *Colour Key*

Supplementary File 3. *Standardized Factor Loadings and Reliability Estimates for Bayes Two-Bifactor of the Self-Compassion Scale Short Form (SCS-SF)*

		PLWCP (N=240)			Community Sample (N=256)		
	**Item No.**	**CSR**	**UCSR**	**r^2**	**CSR**	**UCSR**	**r^2**
**Self-kindness**	2	.75	*-.05*	.61	.63	*.03*	.57
	6	.68	*.13*	.68	.50	*-.02*	.44
**Common humanity**	5	.69	*-.12*	.78	.76	*.02*	.71
	10	.60	*.04*	.56	.57	*-.06*	.41
**Mindfulness**	3	.69	*-.06*	.50	.60	*.01*	.51
	7	.56	*.06*	.54	.59	*.05*	.70
**Self-judgment**	11	*.00*	.80	.72	*-.03*	.80	.74

	12	*.01*	.59	.68	*-.00*	.59	.47
**Isolation**	4	*.06*	.61	.70	*-.01*	.47	.63
	8	*-.02*	.80	.50	*-.08*	.29	.40
**Over-identification**	1	*-.01*	.79	.68	*.05*	.73	.67
	9	*.01*	.75	.66	*.03*	.79	.74

Note. Variable information: PLWCP=people living with chronic pain sample; Community=community sample; CSR=Compassionate Self-Responding (general factor); UCSR=Uncompassionate Self-Responding (general factor). r^2 = item reliability, i.e. percent of variance in the item that is accounted for by the latent factors. Non-significant parameters ($p \geq .05$) are italicized and in grey. All loadings for specific factors were non-significant, except for isolation items 4 and 8 (loadings of .62 and .55 respectively), and self-judgment item 12 (.17) in the community sample, and self-judgment items 11 and 12 (.31 and .41) in the pain sample. All loadings for specific factors were less than .32, except for isolation items 4 and 8 (.62 and .55), self-kindness items 2 and 6 (.32 and .38) and mindfulness item 7 (.51) in the community sample, and common humanity item 10 (.32), mindfulness item 3 (.44), self-judgment item 12 (.41) and isolation item 4 (.51) in the pain sample.
